# Ablation of Complete Staphaloma of the Cornea

**Published:** 1873-08

**Authors:** L. De Wecker


BUFFALO

### Medical and Surgical Journal.

VOL XIII. AUGUST, 1873. No. 1.

### Original Communications.

:o:

ART I.—*Ablation of Complete Staphyloma of the Cornea.* By L. De Wecker. Annals d'Oculistique for January and February, 1873. Translated by H. Norton, M. D., of Detroit, Mich.

The sclerotic suture recommended by Mr. Critchett for ablation of complete staphyloma of the cornea presents in many respects regretable inconveniences. For instance, it has several times occurred to me to be obliged in patients with whom the cicatrization had progressed satisfactorily to have recourse to a secondary operation, consisting in the ablation of points formed at the angle of the linear cicatrix. In reality, the plate of enamel rubbing against these asperities had caused an irritation such that the substitute (artificial eye) had become intolerable to the patients. It is probably due to this secondary operation that I have never passed through that disagreeable experience (which have some English confreres), that the usage of an artificial eye worn upon a stump so irregularly formed is capable of provoking a sympathetic inflammation of the sound eye. Nevertheless, there is no doubt that, in quite young subjects, children of one or two years, in whom the sclerotic is very soft and very elastic, the cicatrization of the suture of Mr. Critchett leaves nothing to be desired in regard to the smoothness of the stump; but it is no longer so in adults, especially when the ectasis is not really limited by the border of the cornea. Some years since Dr. Knapp published observations upon a case where the occlusion of the wound resulting from the ablation of a staphyloma had been effected by him by means of a conjunctival suture. Our esteemed confrere endeavors in passing the needle through the conjunctiva to seize as much episclerotic tissue as he can, so that the conjunctiva slide as little as possible on the sclerotic, and draw this last toward the middle of the palpebral opening as much as it may yield to this traction, thus closing the wound in a satisfactory manner. Evidently Dr. Knapp endeavors to close the wound made by the ablation of the staphyloma in imitation of the method of Mr. Critchett, and to put the lips of the wound in direct contact; but in spite of his two sutures very ingeniously placed this should seem to succeed with him only very imperfectly, if we take into account the following reflection:

"The reunion of the wound," says Dr. Knapp, "was entirely similar to that which results from ablation by the method of Mr. Critchett, only the lips were not completely coaptated longitudinally, but a little folded and gathered, analagous to the folds of a tobacco-pouch." By the operative method which I have to present, I pursue quite another object than do my esteemed confreres, Drs. Critchett and Knapp. I endeavor to arrive at a sub-conjunctival cicatrization of the wound, protected from the contact of air, by closing the lips of the wound in a nonforcible manner, and by avoiding as much as possible all pressure on the globe of the eye. My object is not at all to coaptate the lips of the wound by sutures traversing the sclerotic (Critchett) or the episclerotic tissue (Knapp), which is never possible without exercising a certain pressure, whence results the expulsion of a quantity, more or less considerable, of the vitreous body. I endeavor simply to obtain a reunion as perfect as possible of the conjunctiva over the lips of the wound and of the hyaloid fossa. It appears to me particularly important, in little children, to reduce the globe only to a point to permit a convenient adaptation of an artificial eye. The occasion is presented, unhappily only too frequent, to observe that a considerable diminution of the ocular globe in very young subjects exercises a most injurious influence upon the development of the orbital cavity and upon the growth of all the bones of the corresponding part of the face. All that side of the face remains behind in its development, and attracts by its disproportion the eye of the observer, in despite of the perfect preservation and very satisfying mobility of the artificial piece. It would be assuredly ungraceful in me not to declare openly that the operative procedures of my confreres, Drs Critchett and Knapp, have inspired my method of operation, but this does not the less essentially differ. It is distinguished from Mr. Critchett's method in that I hesitate to pass sutures through the most sensitive portion of the eye, the ciliary body, the sojourn of these sutures, prolonged sometimes during some weeks, necessarily exercising an influence favorable to the shrinking of the eye. The advantage that I obtain by my method over that of Dr. Knapp consists in an occlusion of the wound much more complete, and that without pressure of the vitreous body. For myself, there can be no doubt that my operative method gives a stump the best and the most regular that can be desired for substitution, at the same time that the cure is effected in a manner the most rapid and the least painful to the patient. It has happened to me only once, in a great number of operations, to see arise a suppurative choroiditis. Yet I ought here to observe that the patient had quitted my clinic on the second day, contrary to my wishes, and had attended to his usual occupations without disturbing himself about the precautions which were necessary to the cure of his eye. This is my manner of proceeding: The patient being well asleep, I place by preference a large speculum between the lids, in order to enlarge as much as possible the operative field. I then carefully divide the conjunctiva entirely around and near the border of the cornea, endeavoring, by allowing the scissors to glide between the mucous membrane and the sclerotic, to detach the first as much as I can towards the equator of the eye. This done, I apply four sutures, seizing first the conjunctiva under the border of the cornea and piercing it from without inwards. With the same needle I then perforate the conjunctiva above the border of the cornea at an equal distance from the angle of the wound, penetrating this time the mucous membrane from within outwards, emerging a line or so from the border of the wound. That portion of the sutures between the superior and inferior borders of the cornea over the staphyloma I draw aside—two towards the temple, and two upon the bridge of the nose, in order to avoid the cutting of them in making the ablation of the staphyloma. This ablation is executed by passing a Graefes knife horizontally through the base of the staphyloma, and the section is terminated by detaching the staphyloma very exactly near the border of the cornea, with the aid of scissors, in two semilunar flaps. If the expulsion of the crystalline is not effected spontaneously, I divide the capsule with the cystitome and proceed then immediately to the closure of the sutures. It is well to choose for these sutures silk of different colors, in order to readily perceive the corresponding thread, and also to use very fine English silk to avoid being obliged to preoccupy one's self with taking away the sutures. By the easy gliding of the conjunctiva I obtain a very exact occlusion of the wound, and it is easy for me to avoid, with patients well chloroformed, the least loss of the vitreous body. If I perceive this last tending to form a hernia between the sutures, I place at this point a fifth suture. The cure is effected under the compress bandage, without the least pain, in from ten to fifteen days, leaving an ocular globe sufficiently smooth, and very proper, by its regularity, to serve as a support to the plate of enamel. Without contradiction, it is to perform an act of courage to recommend another new operative method for the ablation of staphyloma, after the number of processes already known. This is particularly applicable to him who pretends to give counsel truly practical, and who does not wish to run the risk of seeing his method fall into a merited oblivion as soon as the attraction of its novelty is passed. That which has principally engaged me to make known my method of operation is the great number of very satisfying cures that it has given me, though I have had many times to record inconveniences after other processes, principally after that of Mr. Critchett. For the rest, the operation which I have described recommends itself principally to those confreres who operate before a numerous public, a circumstance in which the precision and the elegance of a process, at the same time that the security of the patient is complete, are certainly not to be altogether disdained.

